# Worldwide Emergence of Extensively Drug-resistant Tuberculosis

**DOI:** 10.3201/eid1303.061400

**Published:** 2007-03

**Authors:** N. Sarita Shah, Abigail Wright, Gill-Han Bai, Lucia Barrera, Fadila Boulahbal, Nuria Martín-Casabona, Francis Drobniewski, Chris Gilpin, Marta Havelková, Rosario Lepe, Richard Lumb, Beverly Metchock, Françoise Portaels, Maria Filomena Rodrigues, Sabine Rüsch-Gerdes, Armand Van Deun, Veronique Vincent, Kayla Laserson, Charles Wells, J. Peter Cegielski

**Affiliations:** * Centers for Disease Control and Prevention, Atlanta, Georgia, USA; † World Health Organization, Geneva, Switzerland; ‡ Korean Institute of Tuberculosis, Seoul, Republic of Korea; § National Institute of Infectious Diseases, Buenos Aires, Argentina; ¶ Institut Pasteur d’Algérie, Alger, Algeria; # Hospital Universitaris Vall d’Hebron, Barcelona, Spain; ** Health Protection Agency, London, United Kingdom; †† Prince Charles Hospital, Brisbane, Queensland, Australia; ‡‡ National Institute of Public Health, Scrobarova, Czech Republic; §§ Institute of Public Health of Chile, Providencia Santiago, Chile; ¶¶ Institute of Medical and Veterinary Science, Adelaide, South Australia, Australia; ## Institute of Tropical Medicine, Antwerp, Belgium; *** National Institute of Health, Porto, Portugal; ††† National Reference Center for Mycobacteria, Borstel, Germany; ‡‡‡ Institut Pasteur, Paris, France; 1 Current affiliation: Albert Einstein College of Medicine, Bronx, New York, USA; 2 Member: World Health Organization/International Union against Tuberculosis and Lung Diseases Network of Supranational Reference Laboratories

**Keywords:** Mycobacterium tuberculosis, tuberculosis, multidrug-resistant, infectious diseases, emerging, second-line drugs, research

## Abstract

*Mycobacterium tuberculosis* strains that are resistant to an increasing number of second-line drugs used to treat multidrug-resistant tuberculosis (MDR-TB) are becoming a threat to public health worldwide. We surveyed the Network of Supranational Reference Laboratories for *M. tuberculosis* isolates that were resistant to second-line anti-TB drugs during 2000–2004. We defined extensively drug-resistant TB (XDR-TB) as MDR-TB with further resistance to ≥3 of the 6 classes of second-line drugs. Of 23 eligible laboratories, 14 (61%) contributed data on 17,690 isolates, which reflected drug susceptibility results from 48 countries. Of 3,520 (19.9%) MDR-TB isolates, 347 (9.9%) met criteria for XDR-TB. Further investigation of population-based trends and expanded efforts to prevent drug resistance and effectively treat patients with MDR-TB are crucial for protection of public health and control of TB.

## Abstract 2

*Mycobacterium tuberculosis* strains are becoming resistant to not only the most powerful first-line drugs but also many second-line drugs.

Multidrug-resistant tuberculosis (MDR-TB) has been documented in nearly 90 countries and regions worldwide (*1*); 424,203 cases of MDR-TB were estimated to have occurred in 2004, which is 4.3% of all new and previously treated TB cases (*2*). Treatment for MDR-TB patients requires use of second-line drugs for ≥24 months. These drugs are more costly, toxic, and less effective than first-line drugs used for routine treatment of TB (*3*–*6*). As with other diseases, resistance to TB drugs results primarily from nonadherence by patients, incorrect drug prescribing by providers, poor quality drugs, or erratic supply of drugs (*7*).

To facilitate treatment of MDR-TB in resource-limited countries, where most TB cases occur (*1*,*2*), the World Health Organization (WHO) and its partners developed the Green Light Committee, which helps ensure proper use of second-line drugs, to prevent further drug resistance (*8*). Nonetheless, the Green Light Committee encountered numerous anecdotal reports of MDR-TB cases with resistance to most second-line drugs. Once a strain has developed resistance to second-line drugs, these new TB strains are even more difficult to treat with existing drugs. Untreated or inadequately treated patients are at increased risk of spreading their disease in the community, which could lead to outbreaks in vulnerable populations and widespread emergence of a lethal, costly epidemic of drug-resistant TB, reminiscent of the MDR-TB outbreaks in the early 1990s (*9*–*13*). Therefore, to determine whether these anecdotal reports were isolated events, early evidence of an emerging epidemic, or the occurrence of virtually untreatable forms of drug-resistant TB that had not been described previously in different parts of the world, we characterized and quantified the frequency of second-line–drug resistance in several geographic regions.

We sought to determine the extent to which highly resistant *M. tuberculosis* strains have been identified by the international laboratories that participate in the Network of Supranational Reference Laboratories (SRLs). The SRL Network consists of 25 highly proficient TB laboratories on 6 continents. These laboratories collaborate with national reference laboratories to strengthen culture and drug-susceptibility testing capacity and to provide quality control for the WHO/International Union Against Tuberculosis and Lung Diseases Global Project on Anti-TB Drug Resistance (*14*).

## Methods

### Participants

From November 2004 through November 2005, we surveyed the global SRL Network. All SRL directors were invited to participate during the 2004 annual SRL directors meeting, by individual mailings, and by personal phone calls. Drug-susceptibility testing results were requested for *M. tuberculosis* isolates that had been tested for resistance to first-line drugs and second-line drugs during 2000–2004. Two SRLs were not eligible because they did not test for second-line drugs or tested for <3 classes of second-line drugs.

**Figure 1:**
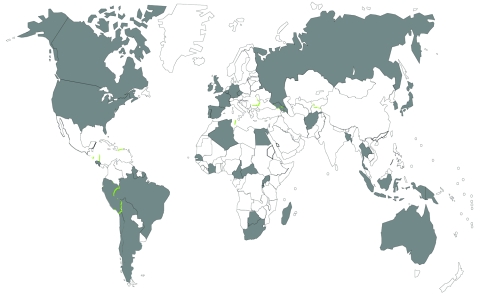
Shading indicates 48 countries that submitted at least 1 isolate to participating Supranational Reference Laboratories, 2000–2004. See Table 4 for complete list of participating countries.

The 14 SRLs that provided data for this study support 112 TB laboratories in 80 countries worldwide (Figure 1). SRLs serve as international reference laboratories to a wide geographic area, performing drug-susceptibility testing that may not be available in a country (e.g., for second-line drugs) and providing quality assurance for first-line–drug testing. Most SRLs also serve as the national reference laboratory for the country in which they are located; they receive varying proportions of isolates from their own and other countries for surveillance, clinical diagnosis, and quality assurance. First-line–drug susceptibility testing is performed on all isolates; second-line–drug susceptibility testing is usually limited to isolates from patients known or suspected to have drug-resistant TB. Of the 14 participating SRLs, not all tested for all 6 classes of second-line drugs, and 4 did not submit data for the entire survey period.

In contrast, the SRL in the Republic of Korea serves as the national reference laboratory and routinely performs an extended diagnostic panel of drug-susceptibility testing on isolates from culture-positive TB patients referred from health centers, hospitals, and clinics in the Republic of Korea. This SRL tests all isolates for 6 classes of second-line drugs; thus, data from the Republic of Korea reflect most culture-positive cases and provide a close approximation to a population estimate of prevalence. Because of the large number of isolates received and because sampling for these isolates is systematically different from that at the other SRLs (testing of all TB patients in the Republic of Korea vs testing of patients more likely to have drug-resistant TB in other SRLs), resistance patterns for the Republic of Korea were analyzed separately from those for the other SRLs.

### Laboratory Methods

Among participating SRLs, different but internationally accepted methods were used to test for second-line drug resistance (details available upon request). Validation of drug-susceptibility testing results for second-line drugs was not performed as part of this survey, but as part of their role as global reference laboratories, all SRLs participate in international proficiency testing for first-line drugs. Quality assurance procedures for second-line–drug susceptibility testing have not been developed; as a proxy for quality assurance, we examined the accuracy of second-line–drug susceptibility testing among isolates susceptible to the 4 main first-line drugs (isoniazid [INH], rifampin [RIF], ethambutol, and streptomycin). On the basis of known mechanisms of drug resistance, finding an isolate that is susceptible to all first-line drugs and resistant to second-line drugs is unlikely (*7*).

### Procedures and Definitions

A standardized reporting form requested anonymous data for all isolates tested for resistance to ≥3 second-line drug classes during 2000–2004. Data were abstracted from the records, electronic or paper, depending on laboratory practices for data management. Results were submitted for 1 isolate per patient. Because SRLs rarely receive multiple isolates from the same patient, reporting of the same patient more than once was unlikely (B. Metchock and G.H. Bai, pers. comm.). No specimens were collected for this study; we used only data from records of isolates that had already been tested. Limited clinical information about the patient was available with each isolate. Consistent data were available for country of origin and date of drug-susceptibility testing. Data about age and TB treatment history were available for <10% of patients, so analysis was not considered reliable for these variables.

To best compare data for the study samples with data from the Global Drug Resistance Survey and other population-based drug-resistance surveillance, we analyzed first-line–drug resistance patterns according to standard methods used in anti-TB–drug resistance surveys (*1*). These patterns included any drug resistance, monoresistance (resistance to only the 1 specified drug), polyresistance (resistance to ≥2 first-line drugs, but which drugs not specified), and multidrug resistance (resistance to at least INH and RIF, with or without other drugs).

We defined 6 classes of second-line drugs as follows: aminoglycosides other than streptomycin (e.g., kanamycin and amikacin), cyclic polypeptides (e.g., capreomycin), fluoroquinolones (e.g., ofloxacin, ciprofloxacin, levofloxacin, and moxifloxacin), thioamides (e.g., prothionamide and ethionamide), serine analogs (e.g., cycloserine and terizidone), and salicylic acid derivatives (e.g., para-aminosalicyclic acid).

For this survey we created a consensus definition that incorporates second-line–drug susceptibility results and is based on international guidelines for management of drug-resistant TB (*15*). The mainstay of an MDR-TB treatment regimen consists of 1 injectible drug (e.g., aminoglycoside or cyclic polypeptide) and a fluoroquinolone; additional drugs from the remaining classes are added until the total reaches 4–6 drugs to which the organism is susceptible. If the infecting organism is resistant to ≥3 second-line drug classes, designing a treatment regimen with sufficient drugs that are known to be effective against TB is difficult. Thus, we defined extensively drug-resistant TB (XDR-TB) isolates as those meeting the criteria established for MDR-TB plus resistance to ≥3 of the 6 classes of second-line drugs.

Second-line–drug resistance patterns were analyzed by geographic region from which the isolate was submitted to the SRL. Regions were grouped into epidemiologically meaningful categories on the basis of prevalence of TB and MDR-TB (*1**,**16*). This retrospective survey was evaluated and approved as public health surveillance by the US Centers for Disease Control and Prevention (CDC).

## Results

**Figure 2:**
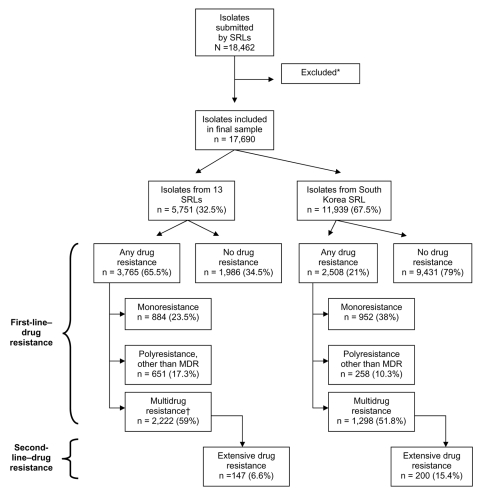
Selection of study sample and summary of drug-resistance patterns of isolates. SRL, Supranational Reference Laboratory. *Tested before 2000 or after 2004 (n = 247) or tested for resistance to <3 classes of second-line drugs (n = 535). †Data for ethambutol resistance missing for 5 isolates.

We received data for 18,462 patients from 14 (61%) of 23 eligible SRLs. We excluded those patients tested before 2000 (n=223), tested after 2004 (n = 14), or tested for resistance to <3 classes of second-line drugs (n = 535). Our final study sample consisted of 17,690 patients whose isolates were tested for resistance to ≥3 second-line drugs during 2000–2004 (Figure 2). Of these, 11,939 (67.5%) patients were from the Republic of Korea and 5,751 (32.5%) were from the remaining SRLs.

### First-line–Drug Susceptibility

Among isolates from patients from the 13 SRLs other than the Republic of Korea, 3,765 (65.5%) were resistant to ≥1 first-line TB drug (Table 1). Of these, 3,305 (58.5%) were resistant to at least INH and 2,345 (41.5%) were resistant to at least RIF. Among isolates from the Republic of Korea patients, 2,508 (21%) had resistance to any drug; most (n = 2,196; 18.4%) were resistant to INH.

**Table 1:** First-line–drug resistance patterns for *Mycobacterium tuberculosis* isolates, 2000–2004 (N = 17,690)*

Pattern	Other 13 SRLs (n = 5,751)		Republic of Korea SRL (n = 11,939)	
	No. tested	No. (%) resistant	No. tested	No. (%) resistant
Any resistance (total)*†‡	5,751	3,765 (65.5)	11,939	2,508 (21.0)
INH	5,645	3,305 (58.5)	11,939	2,196 (18.4)
RIF	5,649	2,345 (41.5)	11,939	1,469 (12.3)
EMB	5,508	1,356 (24.6)	11,939	988 (8.3)
SM	5,618	2,581 (45.9)	11,939	578 (4.8)
				
Monoresistance (total)§¶	5,751	884 (15.4)	11,939	952 (8.0)
INH	5,645	456 (8.1)	11,939	666 (5.6)
RIF	5,649	99 (1.8)	11,939	148 (1.2)
EMB	5,508	8 (0.1)	11,939	25 (0.2)
SM	5,618	321 (5.7)	11,939	113 (0.9)
				
Polyresistance, non-MDR (total)¶	5,644	651 (11.5)	11,939	258 (2.2)
INH + other drugs (except RIF)	5,645	627 (11.1)	11,939	232 (1.9)
RIF + other drugs (except INH)	5,649	24 (0.4)	11,939	23 (0.2)
				
Multidrug resistance (total)¶#	5,644	2,222 (39.4)	11,939	1,298 (10.9)
INH + RIF, only	5,644**	399 (7.1)	11,939	392(3.3)
INH + RIF + EMB, only	5,508**	182 (3.3)	11,939	584 (4.9)
INH + RIF + SM, only	5,618**	619 (11.0)	11,939	89 (0.7)
INH + RIF + EMB + SM	5,476**	1,017 (18.6)	11,939	233 (2.0)

*SRLs, Supranational Reference Laboratories; INH, isoniazid; RIF, rifampin; EMB, ethambutol; SM, streptomycin. †Missing data for INH (106 isolates), RIF (102 isolates), EMB (243 isolates), SM (133 isolates). ‡Cells are not mutually exclusive. §Numerator is isolates with resistance to the specified drug and no known resistance to other first-line drugs. Denominator is isolates tested to at least the specified drug in the numerator. ¶Each cell is mutually exclusive. #Denominator is isolates tested for at least INH + RIF. **Denominator is isolates tested for at least the drugs in the specified combination.

Single-drug resistance was found for isolates from 884 (15.4%) patients from the 13 SRLs; 456 (8.1%) of these were resistant to INH and 99 (1.8%) to RIF. Among isolates from patients from the Republic of Korea, 952 (8%) displayed single-drug resistance, 666 (5.6%) to INH and 148 (1.2%) to RIF.

Polyresistance other than MDR-TB was seen for isolates from 651 (11.5%) patients from the 13 SRLs and 258 (2.2%) from the Republic of Korea SRL. Not all SRLs routinely tested for resistance to pyrazinamide.

Multidrug resistance (i.e., MDR-TB) was present in isolates from 2,222 (39.4%) patients from the 13 SRLs and 1,298 (10.9%) from the Republic of Korea. Resistance to all first-line drugs tested (i.e., MDR-TB with additional resistance to ethambutol and streptomycin) was found in isolates from 1,017 (18.6%) patients from the 13 SRLs and 233 (2%) from the Republic of Korea SRL.

### Second-line–Drug Susceptibility

Among patients from the 13 SRLs, resistance to aminoglycosides was detected in 489 (8.7%) isolates and to fluoroquinolones in 298 (5.3%) (Table 2). Among isolates from Republic of Korea patients, resistance was most commonly seen to fluoroquinolones (n = 524, 4.4%) and thioamides (n = 259, 2.2%).

**Table 2:** Second-line–drug resistance patterns for *Mycobacterium tuberculosis* isolates, 2000–2004 (N = 17,690)*†

Pattern	Other 13 SRLs‡		Republic of Korea SRL‡	
	(n = 5,751)		(n = 11,939)	
	No. tested	No. (%) resistant	No. tested	No. (%) resistant
Any resistance	5,751	1,237 (21.5)	11,939	849 (7.1)
Aminoglycosides§	5,620	489 (8.7)	11,939	227 (1.9)
Capreomycin	4,347	197 (4.5)	11,939	122 (1.0)
Fluoroquinolones	5,580	298 (5.3)	11,939	524 (4.4)
Thioamides	5,131	556 (10.8)	11,939	259 (2.2)
Cycloserine	2,715	70 (2.6)	11,939	80 (0.7)
Para-aminosalicylic acid	3,571	262 (7.3)	11,939	403 (3.4)

*SRLs, Supranational Reference Laboratories.  †Not all isolates were tested for each second-line–drug class (with the exception of the Republic of Korea SRL), so results are reported as a proportion of isolates tested to the specified class of drugs. ‡Cells are not mutually exclusive. §Other than streptomycin (e.g., kanamycin, amikacin).

From all SRLs, isolates that were resistant to at least INH and RIF (i.e., MDR-TB; n = 3,520) and tested for susceptibility to ≥3 second-line drugs were combined for analysis of second-line–drug resistance patterns. Resistance to ≥1 class of second-line drug was present in 1,542 (43.8%) MDR-TB patients (Table 3). The most commonly observed patterns were resistance to aminoglycosides (n = 630, 18.3%), fluoroquinolones (n = 673, 19.3%), and thioamides (n = 605, 19.3%).

**Table 3:** Second-line–drug resistance patterns for multidrug-resistant *Mycobacterium tuberculosis* isolates, 2000–2004*†‡

Pattern	No. tested	No. (%) resistant
Any resistance (total)	3,520	1,542 (43.8)
Aminoglycosides (AG)§	3,442	630 (18.3)
Capreomycin (CM)	2,743	279 (10.2)
Fluoroquinolones (FQ)	3,492	673 (19.3)
Thioamides (TA)	3,132	605 (19.3)
Cycloserine (CS)	2,615	141 (5.4)
Para-aminosalicylic acid (PAS)	2,860	450 (15.7)
		
Extensively drug-resistant TB (XDR-TB, total)¶	3,520	347 (9.9)
AG + CM + FQ	2,656	90 (3.4)
AG + CM + TA	2,498	77 (3.1)
CM + FQ + TA	260	50 (19.2)
AG + FQ + TA	3,040	102 (3.4)
AG + FQ + CS	139	39 (28.1)
FQ + TA + PAS	2,505	94 (3.8)

*Tested for ≥3 second-line drug classes; SRLs, Supranational Reference Laboratories.  †Not all isolates were tested for each second-line drug class (with the exception of the Republic of Korea SRL), so results are reported as a proportion of isolates tested to the specified class of drugs. For combination resistance patterns, results are reported as a proportion of isolates tested to all of the classes of drugs in the specific combination. ‡Cells are not mutually exclusive. §Other than streptomycin (e.g., kanamycin, amikacin). ¶XDR-TB, extensively drug-resistant tuberculosis, i.e., multidrug-resistant tuberculosis (resistant to at least isoniazid and rifampin) with additional resistance to ≥3 classes of second-line drugs.

MDR-TB patients whose isolates had further resistance to ≥3 classes of second-line drugs were classified as XDR-TB (Table 3). A total of 347 (9.9%) MDR-TB patients met criteria for XDR-TB. Among XDR-TB patients, combination drug-resistance patterns included 90 (3.4%) with resistance to aminoglycosides, capreomycin and fluoroquinolones; 102 (3.4%) with resistance to aminoglycosides, fluoroquinolones, and thioamides; and 94 (3.8%) with resistance to fluoroquinolones, thioamides, and para-aminosalicyclic acid. Nearly half (n = 167, 48.1%) of all XDR-TB isolates were resistant to all 4 first-line drugs, bringing the total to ≥7 drugs to which the isolate was resistant.

The proportion of XDR-TB patients by region is shown in Table 4. Among the group of industrialized nations, 53 (6.5%) MDR-TB patients met criteria for XDR-TB. Among patients from Russia and Eastern Europe, 55 (13.6%) MDR-TB patients met criteria for XDR-TB. Among patients from the Republic of Korea, 200 (15.4%) MDR-TB patients, who accounted for 1.7% of all *M. tuberculosis* isolates tested, met criteria for XDR-TB. According to the revised Global XDR-TB Task Force definition (www.who.int/mediacentre/news/notes/2006/np29/en/index.html), 234 (6.6%) isolates met criteria for XDR-TB.

**Table 4:** Extensively drug-resistant tuberculosis among multidrug-resistant tuberculosis isolates, by region, 2000–2004*

Geographic region	Total no. isolates tested†	Total MDR-TB patients	Total XDR-TB patients
	N	n (% of all isolates tested)	n (% of MDR-TB patients)
Industrialized nations‡	2,499	821 (32.9)	53 (6.5)
Latin America§	985	543 (55.1)	32 (5.9)
Eastern Europe¶ and Russia	1,153	406 (35.2)	55 (13.6)
Africa and Middle East#	665	156 (23.5)	1 (0.6)
Asia (other than Republic of Korea)**^*^	391	274 (70.1)	4 (1.5)
Republic of Korea	11,939	1,298 (10.9)	200 (15.4)
Total††		3,418	345

*Region from which isolate was submitted to Supranational Reference Laboratory. MDR-TB, multidrug-resistant tuberculosis; XDR-TB, extensively drug-resistant tuberculosis, i.e., multidrug-resistant tuberculosis (resistant to at least isoniazid and rifampin) with additional resistance to ≥3 classes of second-line drugs. †Total no. of isolates tested for resistance to >3 second-line drug classes, including aminoglycosides (amikacin or kanamycin), polypeptides (capreomycin), fluoroquinolones (ofloxacin or ciprofloxacin), thioamides (ethionamide or prothionamide), cycloserine, and para-aminosalicyclic acid. ^‡^United States, Canada, United Kingdom, countries in Western Europe (Ireland, Portugal, Germany, France, Belgium, Spain), Japan, and Australia. ^§^Argentina, Bolivia, Brazil, Chile, Ecuador, Guyana, French Guiana, Peru, Mexico, Guatemala, El Salvador, Costa Rica. ^¶^Republic of Georgia, Czech Republic, Azerbaijan, Armenia. #Afghanistan, Algeria, Egypt, Tunisia, Botswana, Burundi, Cameroon, Central African Republic, Côte d’Ivoire, Djibouti, Madagascar, Rwanda, South Africa, Senegal, Uganda. ^**^Bangladesh, Indonesia, Papua New Guinea, Thailand, East Timor. ††For 2 XDR-TB patients, data were missing about geographic region.

In evaluating the accuracy of second-line–drug susceptibility testing, we found that 7 (0.1%) of 11,426 patients fully susceptible to all first-line drugs were resistant to 2 second-line drugs, and 109 (1%) were resistant to 1 second-line drug. Most of these patients were resistant to fluoroquinolones.

## Discussion

This study represents the first assessment of the widespread occurrence of *M. tuberculosis* with such extensive drug resistance as to be nearly untreatable with currently available drugs, according to international guidelines. We provide data on second-line–drug resistance for the largest sample of patients to date, including >5,000 patients from 47 countries, apart from the Republic of Korea. The definition of XDR-TB in this survey is based on WHO guidelines for the programmatic management of drug-resistant TB; the guidelines recommend treatment with ≥4 drugs known to be effective (*15*). Therefore, with ≤3 remaining classes of second-line drugs to which the infecting organism is susceptible, treatment of these patients cannot meet international standards. XDR-TB has been detected in all regions of the world. XDR-TB strains in this study also have high rates of resistance to pyrazinamide and ethambutol, thereby severely limiting the treatment options available.

Analysis of combination second-line–drug resistance patterns is critical for clinicians and policymakers who design treatment regimens for these patients. Although limited data exist in the literature about second-line–drug resistance patterns among MDR-TB patients, data from patients undergoing retreatment for TB in Hong Kong showed that 30 (17%) MDR-TB isolates were resistant to ≥3 second-line drugs (*17*), thereby meeting criteria for XDR-TB. A drug-resistance survey of 447 culture-positive new patients and patients undergoing retreatment in Abkhazia, Republic of Georgia, found that of 63 MDR-TB patients, 2 (3%) had additional resistance to 3 second-line drug classes, consistent with XDR-TB (*18*). More recently, clusters of XDR-TB have been reported in South Africa and Iran (*19*,*20*) and have been associated with HIV infection and rapid and high death rates.

The emergence of new strains of TB that are resistant to second-line drugs, especially in settings where TB control programs have become unable to adequately monitor treatment regimens for MDR-TB, is cause for concern. After the resurgence of TB in industrialized countries during the 1980s and increased awareness of this global problem, implementation of strong TB control programs based on the principles of the global d**irectly observed treatment** strategy, short course (DOTS) improved treatment outcomes and reduced TB and MDR-TB incidence in several countries. This framework for DOTS, promulgated by WHO, and the pilot MDR-TB management projects (DOTS-Plus projects) became the basis for programmatic management of MDR-TB, which has demonstrated feasibility and effectiveness in low- and middle-income countries (*5*,*15*). However, second-line drugs are available worldwide outside of well-organized TB-control programs (WHO, unpub. data).

Improper treatment of drug-resistant TB, such as using too few drugs, relying on poor quality second-line drugs, and failing to ensure adherence to treatment, will likely lead to increases in XDR-TB. Strengthening basic TB programs and infection control measures is crucial for preventing the selective pressure and environments in which resistant strains are transmitted from person to person. Additionally, MDR-TB programs that rely on quality-assured and internationally recommended treatment regimens according to WHO guidelines must be scaled up and strengthened to stem further second-line–drug resistance and spread of XDR-TB. The Green Light Committee provides a global mechanism to help affected countries achieve these steps. A commentary published in 2000 predicted that “failure to institute [the] entire DOTS-Plus package is likely to destroy the last tools available to combat [TB], and may ultimately result in the victory of the tubercle bacillus over mankind” (*21*). XDR-TB is an indirect indicator of program failure to adequately diagnose, prevent, and treat MDR-TB.

Documenting the emergence of XDR-TB requires a laboratory-based diagnosis that relies on first- and second-line–drug susceptibility testing. A limitation to accurate detection of XDR-TB is that existing tests for resistance to second-line drugs are not yet standardized and are less reproducible than tests for resistance to INH and RIF. Lack of international recommendations for use, as well as lack of standardization and the historical unavailability of MDR-TB treatment in the public sector, has limited use of second-line–drug susceptibility testing on a wider scale. As access to treatment with second-line drugs increases, standardized methods, improved diagnostics, and quality assurance for second-line–drug susceptibility testing are urgently needed to enable reliable testing and design of appropriate treatment regimens. Although internationally accepted methods were used by all laboratories, the precise methods and drug concentrations used varied among participating SRLs (*22*). Because these SRLs represent some of the most highly performing laboratories on 6 continents, results of drug-susceptibility testing are credible within the context of stated limitations. Initial studies that standardized different methods for second-line–drug susceptibility testing have been completed (*23**–**26*), but more are needed.

Our study has other limitations. The numbers reported for XDR-TB probably represent an underestimate of the true number of cases because not all SRLs and not all national reference laboratories test for all 6 classes of second-line drugs. In the absence of test results for all 6 classes of second-line drugs, we speculate, on the basis of a patient’s TB treatment history and known patterns of drug cross-resistance, that many other unidentified patients are likely to have had and died from XDR-TB. For example, an MDR-TB isolate that is also resistant to an aminoglycoside and a fluoroquinolone but that has not been tested for the other second-line drug classes is very likely to be resistant to an additional second-line drug class for the following reasons: INH and ethionamide have a 15%–20% rate of cross-resistance (*27*); kanamycin and capreomycin cross-resistance is common, ranging from 20%–60% (CDC, unpub. data) (*28**,**29*); and in this study, isolates that were resistant to all 4 first-line drugs as well as an aminoglycoside and a fluoroquinolone were 70%–80% likely to be resistant to at least 1 additional class of second-line drug.

Another limitation is that data from most SRLs were drawn from a convenience sample of isolates and reflect referral bias. Thus, these data can not be considered representative of a patient population or region, and actual denominators are difficult to determine. For this reason, although estimates of prevalence are possible, they cannot be generalized to the local or regional population. However, our study is the first to report XDR-TB patients in multiple geographic regions; future systematic surveys are needed to determine the true extent of this disease. Data from the Republic of Korea reflect a more comprehensive policy for drug-susceptibility testing and provide an estimate of the population prevalence in this setting. However, the 10.9% rate of MDR-TB for the Republic of Korea is higher than rates reported from other national drug resistance surveys and may reflect other unknown referral biases (*1*).

Lastly, we had limited clinical information about each patient because information submitted to each SRL varied and was not reliably available for inclusion in the analysis. Data about TB treatment history, patient age and sex, or HIV status are not routinely collected by all laboratories. Genotyping data were not available to confirm whether XDR-TB isolates are related to W variant of the Beijing strain, a highly drug-resistant strain of *M. tuberculosis* responsible for large nosocomial outbreaks in New York in the early 1990s (*30*).

Despite these limitations, our survey provides the first documentation of the emergence of XDR-TB as a serious worldwide public health threat. XDR-TB was identified on 6 continents and is significantly associated with worse treatment outcomes than MDR-TB (*31**,**32*). The emergence of XDR-TB, coupled with the increased use of second-line drugs, suggests that urgent measures are needed to improve rational use of quality-assured second-line drugs. In addition, population-based surveillance for second-line–drug susceptibility testing is needed to better describe the magnitude of XDR-TB worldwide, track trends, and plan a public health response. Indeed, the convergence of XDR-TB with the HIV epidemic may undermine gains in HIV prevention and treatment programs and requires urgent interventions. These interventions include ensuring adherence to recommended international standards of care aimed at promptly and reliably diagnosing TB, ensuring adherence to recommended treatment regimens with demonstrated efficacy, implementing infection control precautions where patients congregate, and improving laboratories’ capacity to accurately and rapidly detect drug-resistant *M. tuberculosis* isolates so that patients can receive effective treatment (*33*). Other unmet needs include further development of international standards for second-line–drug susceptibility testing, new anti-TB drug regimens, and better diagnostic tests for TB and MDR-TB. Such measures are crucial if future generations are to be protected from potentially untreatable TB.
